# A comparison of normal and high post-void residual urine and urodynamic parameters in women with overactive bladder

**DOI:** 10.4274/tjod.84666

**Published:** 2017-12-30

**Authors:** Hediye Dağdeviren, Hüseyin Cengiz, Çağlar Helvacıoğlu, Ulkar Heydarova, Cihan Kaya, Murat Ekin

**Affiliations:** 1 University of Health Sciences, Bakırköy Dr. Sadi Konuk Training and Research Hospital, Clinic of Obstetric and Gynecology, İstanbul, Turkey

**Keywords:** Residue urine, urodynamics, voiding, bladder pressure

## Abstract

**Objective:**

To investigate voiding functions and assess the relationships of voiding parameters to overactive bladder symptoms and postvoiding residue volumes.

**Materials and Methods:**

This is a retrospective study analyzing urodynamic parameters in patients who were diagnosed as having overactive in our urogynecology clinic between April 2014 and April 2016. A total of 290 women who met the selection criteria were included in the study. The patients were divided into two groups according to postvoiding residue volumes: group 1, postvoiding residue volumes <100 mL (n=135); group 2, postvoiding residue volumes ≥100 mL (n=155).

**Results:**

A total of 290 women were included in the study; the mean age was 71.4 years. A total of 158 (54.5%) patients had detrusor over-activity during urodynamic testing. The mean maximum bladder capacity in elevated group 2 (postvoiding residue volumes ≥100 mL) was significantly higher than in group 1 (postvoiding residue volumes <100 mL) (p<0.01). Additionally, there was a significant difference between detrusor pressure at Q_max_ in both study groups (p<0.05). There were no significant differences in the first-sensation volume between the normal and elevated postvoiding residue volumes groups.

**Conclusion:**

In conclusion, patients with overactive with elevated postvoiding residue volumes showed increased maximum bladder capacity, but detrusor over-activity was not more prevalent in these women compared with women with normal postvoiding residue volumes.

## PRECIS:

We studied urodynamic parameters and their clinical importance in patients with overactive bladders.

## INTRODUCTION

Urinary incontinence is defined as involuntary leakage of urine^(1,2)^. It is estimated that between 26 and 61% of women receive care for urinary incontinence in their lifetime^(3,4)^. Overactive bladder (OAB) is a term that describes a syndrome of urinary urgency with or without incontinence, often accompanied by nocturia and urinary frequency^(5,6)^.

Urodynamics comprises a group of tests used to evaluate urinary tract function. Urodynamic testing is a simple and non-invasive procedure for evaluating lower urinary tract symptoms. It is also a way of assessing maximum flow rate (Q_maximum_; Q_max_), average flow rate (Q_average_; Q_ave_), bladder capacity, and post-void residual (PVR) urine^(7)^.

Voiding dysfunction is a broad term. It is defined by the International Urogynecological Assosication/International Continence Society as “incomplete micturition or abnormally slow micturition^(8)^.” It is the cause of elevated PVR. Parameters for interpreting the results of PVR testing are neither standardized nor well evaluated. In general, a PVR of less than one-third of total voided volume is considered adequate emptying^(9,10)^. Additional parameters include designation of PVR greater than 100 mL as abnormal^(11)^.

Urodynamic parameters and their clinical importance in patients with OAB have not been well studied. Therefore, we explored voiding functions and assessed the relationships of voiding parameters with OAB symptoms and PVR volumes.

## MATERIALS AND METHODS

The patients’ data were retrospectively collected from the hospital medical records. We analyzed urodynamic parameters in patients who diagnosed as having OAB in our urogynecology clinic between April 2014 and April 2016. All patients signed informed consent forms. The same researchers (H.D. and C.H.) conducted retrospective chart reviews for all patients with OAB. A total of 290 women who met the selection criteria were included in the study. The patients were divided into two groups according to PVR: group 1, PVR <100 mL (n=135); group 2, PVR ≥100 mL (n=155).

OAB syndrome is defined as urinary urgency with or without urge incontinence, typically associated with increased daytime frequency and nocturia^(12)^. Detrusor over-activity (DO) is a urodynamic symptom characterized by involuntary detrusor contractions during the filling phase, which may be spontaneous or provoked^(1)^.

Exclusion criteria were patients with pure stress incontinence, allergic diseases such as asthma, history of psychiatric disorders, urinary tract infection, urinary obstructive disease, metabolic diseases, neurologic disorders, and current use of diuretics.

All urodynamic tests were conducted by the same registered nurse and author (H.C.); the same instructions were used to prepare patients, and the same urodynamic machine was used for testing. Patients were evaluated using a multi-channel MMS® Solar (ADS, Ltd., Enschede, The Netherlands) urodynamic study device. Filling cystometry and uroflowmetry were performed. Also residual urine was measured. Isotonic saline was infused at a rate of 50 mL/min at room temperature for the filling. The patients were in the semi-sitting position. After the urinary bladder was full, volumes at first sensation of urination (mL), feeling the need to urinate (mL), feeling the need to urinate immediately (mL), bladder capacity (mL), and compliance (mL/cm H_2_O) were measured. Detrusor contractions present after 15 cm H_2_O that could not be inhibited were recorded. During the procedure, maneuvers such as coughing and straining were performed to assess whether urinary leakage from the external meatus occurred. The results of urodynamic tests were interpreted and reported by a urogynecologist (H.C.) using an identical reporting form. Catheters for vesical and abdominal pressures were reset before placement of the catheters.

The filling sensations assessed as volumes at first sensation; first urge sensation; strong urge sensation; and bladder capacity. After reaching cystometric bladder capacity, all women were given permission to void. Each woman was repositioned to an upright position. In this position they could void without abdominal pressure. After the measurement of pressures, PVR volume was measured by a nurse who attached a urethral catheter. Bladder capacity was calculated. The values of Q_max_ and detrusor pressure at Q_max_ were calculated from the pressure-flow testing.

### Statistical Analysis

Statistical analysis was performed using the Number Cruncher Statistical System 2007 statistical software. The mean differences between groups were compared using Student’s t-test, and the Mann-Whitney U and independent-samples t-test were applied for comparisons of median values. Logistic regression analyses were conducted to determine the association between voiding parameters and PVR volume. A p value less than 0.05 was considered statistically significant.

## RESULTS

A total of 290 women were included in the study; the mean age was 71.4 years. Table 1 shows the characteristics of the women. A total of 158 (54.5%) patients had DO during urodynamic testing. The mean maximum bladder capacity in elevated group 2 (PVR ≥100 mL) was significantly higher than in group 1 (PVR <100 mL) (p<0.01). Additionally, there was a significant difference between detrusor pressure at Q_max_ in both study groups (p<0.05). There were no significant differences in first-sensation volume between the normal and elevated PVR groups. Women with elevated PVR had significantly higher voided volume and maximum flow rates than did women with normal PVR. Additionally, we found no significant difference in DO between the two groups (p=0.282). Table 2 shows a comparison of urodynamic parameters through PVR volumes. Table 3 shows the results of logistic regression analysis to determine the best predictor(s) to discriminate between the groups.

## DISCUSSION

Urodynamic evaluation is a non-invasive process that provides objective and subjective data about bladder functioning. Measurements of urine flow and PVR with uroflowmetry are important parameters. There are some parameters that affect the urine flow rate including voided volume, detrusor contractibility, and bladder outlet obstruction. The purpose of this study was to evaluate urodynamic parameters in women who had OAB symptoms and either normal or elevated PVR volumes. There were 290 medical records for the analyses in the clinical setting.

During urodynamic testing, 158 (54.8%) women showed DO. In this study we find that nearly 50% of patients with OAB had DO on urodynamics testing. Our results are compatible with previous studies^(11,13,14)^. Additionally, Futyma et al.^(15)^ observed DO in less than 20% of patients with OAB, which is much lower than the values in other studies where DO was found in more than 50% of patients. The authors believed that the difference in DO prevalence was associated with patients’ performing maneuvers during the test that may have provoked urinary responses. The differences among studies may reflect differences in patient selection criteria before data analysis. Jeong et al.^(16)^ observed higher Q_max_ detrusor pressures than were found in our study. We hypothesized that the difference might be explained by the age differences of the study samples. The mean age in the present study was 71 years, whereas in Jeong et al.^(16)^ the mean age was 58.9 years. It has been established that bladder and uretral profiles change with age. Women who had voided less than 100 mL (n=18) were excluded from the present study because the minimum voided volume for reliable urine flow rate in persons aged 56-80 years is 100 mL^(17)^.

PVR volume can be measured using uroflowmetry or pressure-flow studies. These tests are non-invasive. Previous reports reflected a debate regarding the measurement of residual urine volume. Specifically, when voided volume was greater than 300 mL, the mean PVR volumes were not significantly different between non-invasive uroflowmetries and pressure flow studies^(18)^. Therefore, further studies on the differences in PVR volume in relation to voided volume and type of testing are required. Other limitations of the current study are related to its retrospective design. We did not use a bladder diary data to examine the women’s naturally voided volume.

## CONCLUSION

Patients with OAB with elevated PVR volumes showed increased maximum bladder capacity, but DO was not more prevalent in these women compared with women with normal PVR volumes.

## Figures and Tables

**Table 1:**
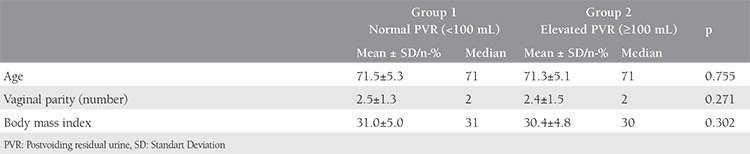
Characteristics of the study groups

**Table 2:**
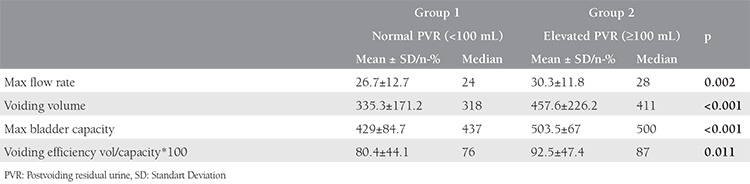
Comparison of urodynamic parameters through post-void residual volumes

**Table 3:**
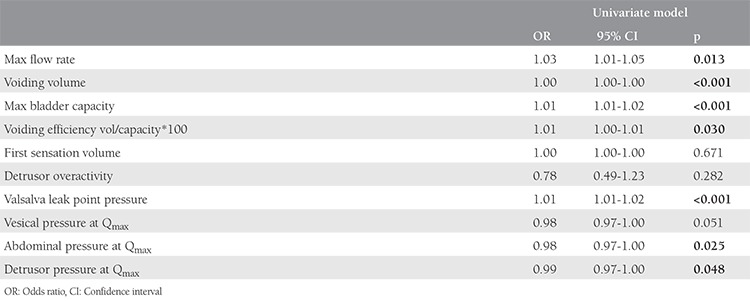
Logistic regression analysis of voiding parameters though post-voidal residual urine in patients with overactive bladder
